# 2D/1D MXene/MWCNT Hybrid Membrane-Based Evaporator for Solar Desalination

**DOI:** 10.3390/ma15030929

**Published:** 2022-01-25

**Authors:** Yawei Yang, Yan Han, Jianqiu Zhao, Wenxiu Que

**Affiliations:** Electronic Materials Research Laboratory, Key Laboratory of the Ministry of Education, International Center for Dielectric Research, Shaanxi Engineering Research Center of Advanced Energy Materials and Devices, School of Electronic Science and Engineering, Xi’an Jiaotong University, Xi’an 710049, China; hanyan0302@stu.xjtu.edu.cn (Y.H.); jqzhao1994@163.com (J.Z.)

**Keywords:** MXene, MWCNT, hybrid membrane, solar desalination

## Abstract

Solar vapor generation through evaporation using photothermal materials is a promising candidate for seawater desalination. The Ti_3_C_2_ MXene membrane has exhibited photothermal behavior in solar water evaporation. However, dense packed two-dimensional (2D) MXene membrane with high reflection loss and insufficient vapor escape channels limited its solar evaporation performance. In this work, one-dimensional (1D) multi-walled carbon nanotubes (MWCNT) were added into 2D Ti_3_C_2_ nanosheets as the holder to form a 2D/1D hybrid photothermal membrane. Owing to the 2D/1D hybrid structure, more effective broadband solar absorption, water transportation and vapor escape were achieved.

## 1. Introduction

Extracting freshwater from saline water sources, known as the desalination, is a common way to solve the freshwater shortage issue all over the world. Among various desalination technologies, vapor generation from seawater driven by solar energy has become one of the most promising strategies for clean water harvesting [1]. Solar vapor generation technology is not only for seawater desalination, but also can be used for wastewater treatment and electricity generation [2,3].

Although several examples of photothermal membranes, including carbon-related materials [4,5], narrow-bandgap semiconductors [6,7,8] and plasmonic nanoparticles [9,10], have been investigated for solar desalination, two-dimensional (2D) Ti_3_C_2_ MXene nanosheets with broadband spectrum absorption and an 100% internal light-to-heat conversion efficiency have drawn intensive attention in this area [11,12]. 2D photothermal membranes located at the water-air interface have exhibited vapor generation capability and low heat loss into the bulk water. However, the pristine 2D nanosheets membrane is dense stacked, leading to a high light reflection and limited pathway for water molecules passing through, which seriously hinders solar evaporation performance [13,14]. Various morphologies of Ti_3_C_2_ nanomaterials have been reported for improving solar desalination performance, including aerogel, foam, hydrogel, membrane, monoliths, porous and nanocomposite [15]. For 2D Ti_3_C_2_ nanosheets-based photothermal membranes, zero-dimensional (0D) Au nanoparticles, 2D MoS_2_ nanosheets and three-dimensional (3D) Cu_3_BiS_3_ nanoflowers were intercalated into 2D Ti_3_C_2_ layers to form 2D/0D Ti_3_C_2_/Au [16], 2D/2D Ti_3_C_2_/MoS_2_ [17] and 2D/3D Ti_3_C_2_/Cu_3_BiS_3_ [18] composite membranes, respectively. Hence, photothermal membrane with rough surface and porous structure by intercalating various dimensional nanostructures has become a candidate for promoting water transportation and vapor escape. Adding one-dimensional (1D) multi-walled carbon nanotubes (MWCNT) with excellent hydrophilicity and thermal conductivity into the 2D membrane is also considered as an effective strategy for enhancing the porosity, light absorption and thermal conduction to the interfacial water. In this work, the 2D/1D MXene/MWCNT hybrid membrane was fabricated as the solar absorber for enhancing the vapor generation and seawater desalination performance.

## 2. Materials and Methods

### 2.1. Materials

Al (1–3 μm, 99.5%), TiC (2–4 μm, 99%) and Ti (≤48 μm, 99.99%) powders were purchased from Aladdin. Hydrochloric acid (HCl, 36–38%), sulfuric acid (H_2_SO_4_, 95–98%), nitric acid (HNO_3_, 65–68%) and ethanol (99.7%) were provided by Sinopharm Chemical. Multi-walled carbon nanotubes (MWCNT) were purchased from Beijing Deke Daojin Science and Technology Co., Ltd., Beijing, China. Hydrophilic mixed cellulose ester filter membrane (0.22 μm in pores size, 5 cm in diameter) was purchased from Shanghai Xinya Purification Equipment Co., Shanghai, China. All chemicals were used as received without further purification.

### 2.2. Preparation of Ti_3_C_2_ MXenen Nanosheets Suspension

The mixture of TiC, Al and Ti powders with a molar ratio of 2.0:1.2:1.0 was ball-milled with ethyl alcohol for 4 h at 300 rpm, and dried in vacuum at 60 °C. Ti_3_AlC_2_ was prepared by annealing the mixture to 1350 °C for 2 h in Ar flow. The sintered product was grinded and sieved through a 400 mesh screen, so that the particle size was controlled <38 μm. Ti_3_C_2_ nanosheets were prepared by etching away the Al layer of Ti_3_AlC_2_ with etching solution. The etching solution was obtained by slowly dissolving 2 g LiF in 15 mL 9 M HCl under stirring for 5 min. Then, 1 g Ti_3_AlC_2_ powder was slowly added into the etching solution at 35 °C under stirring for 24 h. The product was washed with water and centrifuged at 3500 rpm for 2 min for several times until pH > 6. After the final cycle, the Ti_3_C_2_ precipitate was dispersed in 200 mL water, followed by degassing and N_2_ bubbling for 1 h. Finally, the suspension was centrifuged at 3500 rpm for 30 min to remove the unetched ingredients, and the supernatant was the exfoliated monolayer or few-layer Ti_3_C_2_ nanosheets suspension.

### 2.3. Preparation of MWCNT Suspension

The original MWCNT was treated by acid to improve its hydrophilicity. First, 60 mL H_2_SO_4_ was slowly added into 20 mL HNO_3_ under stirring. Then, 1 g original MWCNT was slowly added into the mixed acid, and the mixture was heated to 80 °C for 2 h under stirring. The mixture was centrifuged at 10,000 rpm for 10 min, and the product was washed with water for several times. Finally, the acidified MWCNT was well-dispersed in water to form MWCNT suspension.

### 2.4. Fabrication of the Ti_3_C_2_-MWCNT Membrane-Based Evaporator

**Figure 1:**
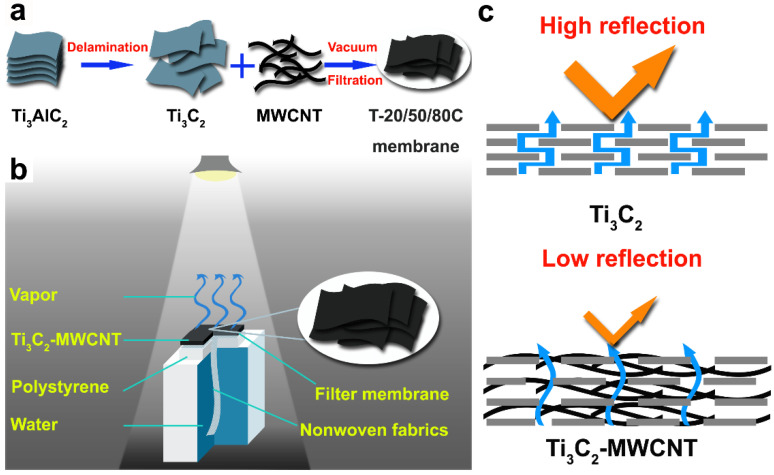
Scheme of (**a**) fabrication processes of the Ti_3_C_2_-MWCNT membranes, (**b**) the solar evaporator and (**c**) light reflection and vapor flux.

Various amount of MWCNT suspension (0, 20 wt%, 50 wt% and 80 wt%) was added into Ti_3_C_2_ nanosheets suspension under stirring, and the total mass of the two components was fixed to 10 mg. After mixing uniformly, the mixed suspension was vacuum filtered on a hydrophilic mixed cellulose ester filter membrane with a diameter of 4 cm, followed by drying at 40 °C to form a Ti_3_C_2_-MWCNT membrane (Figure 1a). The corresponding sequential Ti_3_C_2_-MWCNT membranes were recorded as Ti_3_C_2_, T-20C, T-50C and T-80C, respectively. The Ti_3_C_2_-MWCNT membrane-based evaporator was fabricated according to our previous work (Figure 1b) [14], consists of three parts: (i) a piece of Ti_3_C_2_-MWCNT membrane as the solar absorber and vapor generator, (ii) a polystyrene foam as the thermal insulator and floater and (iii) a non-woven fabric as the water transportation pathway by capillary effect.

### 2.5. Characterizations

The crystal phase was analyzed by a powder X-ray diffraction spectroscopy (XRD, SmartLab, Rigaku, Akishima, Tokyo, Japan) with Cu Ka radiation (40 kV, 30 mA). The microstructure was characterized by scanning electron microscopy (SEM, Quatan250 FEG, FEI, Hillsboro, OR, USA) and transmission electron microscopy (TEM, JEM-2100F, JEOL, Akishima, Tokyo, Japan). The UV-Vis-NIR absorption spectrum was measured by a UV/Vis/NIR spectrometer (PerkinElmer, Lambda 950, Waltham, MA, USA). A contact angle meter (JC2000D5, Powereach, Shanghai, China) was used to characterize the wettability. The salinity of water before and after solar evaporation was tracked by an inductively coupled plasma spectroscopy (ICP-OES, PerkinElmer Optima 8000, Waltham, MA, USA).

### 2.6. Solar Vapor Generation Measurement

The membrane was cut into a size of 2 × 2 cm^2^. The evaporator was self-floated on the water in a Teflon container under the simulated solar illumination (Newport Oriel, 1.0 kW/m^2^) at a temperature of 25~26 °C and a humidity of 30~40%. The temperature was measured by an IR camera (Fluke, VT04A, Everett, WA, USA). The water weight loss through evaporation was measured by an electronic analytical balance (0.1 mg in accuracy). The corresponding solar-to-vapor conversion efficiency can be calculated by the evaporation rate derived from weight loss curve (see Supporting Information for details). The seawater desalination was performed by using natural seawater (Bohai Sea, salinity ~2.75%).

## 3. Results and Discussion

**Figure 2:**
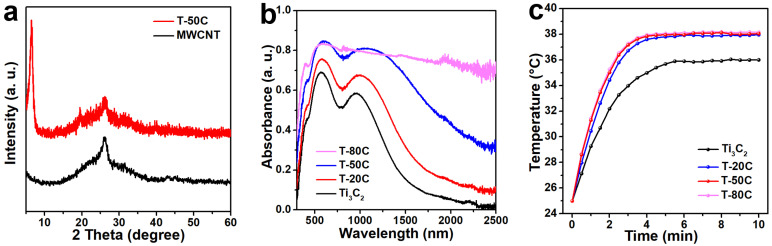
(**a**) XRD patterns, (**b**) UV-Vis-NIR absorption spectra and (**c**) temperature variation of the vapor over time of the Ti_3_C_2_-MWCNT membranes.

The MWCNT and Ti_3_C_2_ nanosheets suspensions are stable homogeneous colloidal dispersions with particle size <100 nm (Figures S1 and S2), which is benefit for membrane preparation. Two diffraction peaks at 2θ ≈ 6° and 26° in XRD patterns can be indexed to (002) planes of Ti_3_C_2_ nanosheets and (002) planes of the graphite phase of MWCNT, respectively (Figure 2a). This result suggests that the Ti_3_C_2_ nanosheets remains 2D structure in the hybrid membrane. Besides, the hydrophilicity of the membrane has a positive impact on water transportation [19], and it improves with the increasing MWCNT content due to the adsorbed hydroxyl and carboxyl groups on MWCNT surface during acidification (Figure S3). The absorption of the Ti_3_C_2_-MWCNT hybrid membranes covers the entire solar spectrum region (Figure 2b). Two absorption peaks at ~600 nm and ~1000 nm can be assigned to the intrinsic absorption of the 2D Ti_3_C_2_ membrane [14], which are almost covered by MWCNT absorption in T-80C due to the high MWCNT content. A strong light reflection can be seen in the 2D Ti_3_C_2_ membrane due to its smooth surface (Figure S4a,b). An increased light absorption is achieved for the hybrid membranes compared to the pristine Ti_3_C_2_ membrane, which should be attributed to the presence of MWCNT increasing light absorption and decreasing light reflection of the membrane (Figure S4c,d). As a result, the vapor temperature of all the hybrid membrane can rapidly reach to a steady high value of ~38 °C within 4 min upon solar irradiation (Figure 2c), which is due to the local heating of the membrane and a limited conduction loss of the thermal insulating layer. The vapor temperature rises a bit faster with the increasing MWCNT content in first 4 min. However, for the pristine Ti_3_C_2_ membrane, a lower steady vapor temperature of ~36 °C is achieved in a longer time of 6 min.

**Figure 3:**
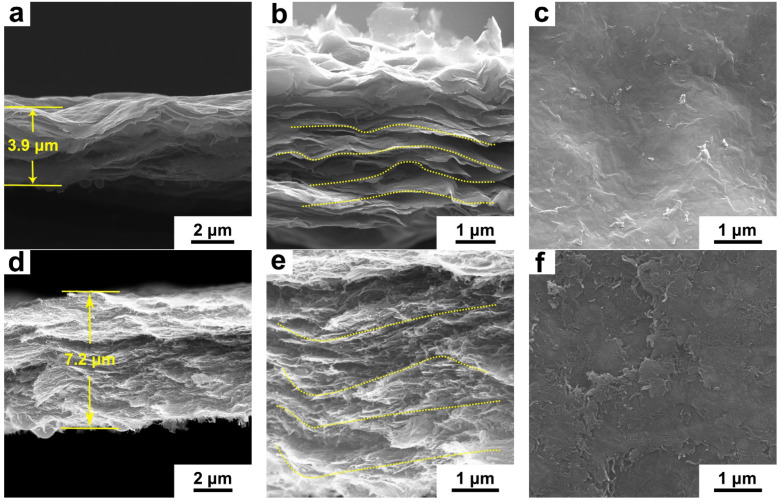
(**a**,**b**,**d**,**e**) Cross-section and (**c**,**f**) top-view SEM images of the Ti_3_C_2_/T-50C membranes.

The Ti_3_C_2_ membrane exhibits a clear layered structure with a thickness of ~3.9 μm (Figure 3a,b) and a dense flat surface (Figure 3c), due to the compact stacking of the 2D nanosheets. For hybrid membranes, 1D MWCNT uniformly intercalates between the 2D Ti_3_C_2_ nanosheets as the holder, which enlarges the gaps between the layers, leading to a loose structure. As a result, with the increase of MWCNT content, the thickness of the hybrid membranes significantly increases to ~7.7, 7.2 and 6.9 μm for T-20C, T-50C and T-80C, respectively (Figure 3d and Figure S5a,d). The thicker composite layer indicates a loose structure of the membrane under the fixed total materials. The thickness of the Ti_3_C_2_-MWCNT hybrid membrane nearly doubles compared to pristine Ti_3_C_2_ membrane due to the interlaminated 1D MWCNT, but slightly decreases with the increase of MWCNT content because the layered structure fades away with the reducing nanosheets component, which compacts the membrane again (Figure 3d and Figure S5a,d). The larger gaps between 2D layers (Figure 3b,e) and distinct pores in the surface (Figure 3f) formed by 1D MWCNT support inside the photothermal membrane promote the water transportation and vapor escape during solar evaporation [13].

**Figure 4:**
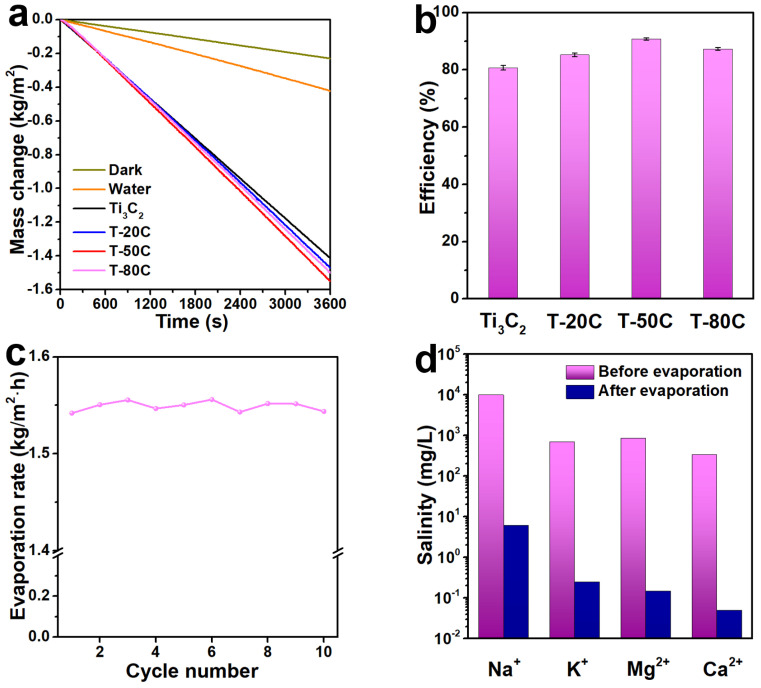
(**a**) Water weight loss through solar evaporation, (**b**) the corresponding solar-to-vapor conversion efficiency, (**c**) cycle running and (**d**) salinity before and after solar desalination.

The average evaporation rate of water, Ti_3_C_2_, T-20C, T-50C and T-80C membranes under one Sun irradiation is 0.42, 1.41, 1.47, 1.55 and 1.50 kg/m^2^·h, respectively (Figure 4a). The optimized solar evaporation rate is 3.7 times higher than that of pristine water. The dark evaporation rate (natural volatilization) of all these membranes is 0.23 kg/m^2^·h. Therefore, the net evaporation rate is 1.18, 1.24, 1.32 and 1.27 kg/m^2^·h, respectively. Hence, the average solar-to-vapor conversion efficiency (*η*) is calculated by Equations (S1)–(S4) (Supporting Information) to be 80.7%, 85.3%, 90.8% and 87.3%, respectively (Figure 4b), superior to most of the previously reported 2D materials-based photothermal membranes (Table S1). Obviously, when 1D MWCNT was introduced into the 2D Ti_3_C_2_ membrane, the evaporation rate and conversion efficiency of the hybrid membrane are significantly improved, which can be attributed to the enhanced light absorption and water molecules transportation of the Ti_3_C_2_-MWCNT hybrid membrane (Figure 1c). Although the light absorption ability of the hybrid membrane is improved with the increase of MWCNT content, the conversion efficiency increases firstly, and then decreases as the increase of MWCNT content. In other words, the T-50C membrane exhibits the highest solar evaporation performance, which indicates that the optimized 2D/1D structure is benefit for water transportation and vapor escape. It can be seen that there is an optimized membrane thickness for solar evaporation. Because too thin membrane cannot fully absorb the sunlight, but too thick one may loss heat during downward conduction. Therefore, there is a balance between the light absorption and heat conduction for the highest heat utilization. In the cycle running of the T-50C membrane with each cycle lasting for 12 h, the solar evaporation rate is basically located in the range of 1.54~1.56 kg/m^2^·h with an average value of 1.55 kg/m^2^·h without decay (Figure 4c), suggesting the stability of the hybrid membrane. To evaluate the desalination performance of the membrane, the salinity before and after solar evaporation was tracked. The concentration of four primary ions (Na^+^, K^+^, Mg^2+^ and Ca^2+^) is blocked over 99.5% (Figure 4d), outclassing the drinking standard. These results suggest that the 2D/1D Ti_3_C_2_-MWCNT hybrid membrane-based evaporator is a good candidate for solar desalination.

## 4. Conclusions

1D MWCNT was added into 2D Ti_3_C_2_ nanosheets as the holder to form a hybrid photothermal membrane, which acts as the light absorber and vapor evaporator. An optimized solar evaporation rate of 1.55 kg/m^2^·h, and corresponding solar-to-vapor conversion efficiency of 90.8% were achieved over the T-50C membrane, in which the light absorption, water transportation and vapor escape capabilities were significantly enhanced compared to the pristine 2D Ti_3_C_2_ membrane. The present study is expected to provide a rational design for enhancing the solar vapor generation performance of the 2D membrane-based evaporators.

## Data Availability

The data supporting reported results of the current study are available from the corresponding authors on reasonable request.

## Supplementary Materials

The following supporting information can be downloaded at: https://www.mdpi.com/article/10.3390/ma15030929/s1, Figure S1: Photographs of (a) the original MWCNT after 3 h of ultrasonic dispersion (left) and well-dispersed acidified MWCNT suspension (right), (b) no Tyndall effect in original MWCNT, (c) Tyndall effect in MWCNT suspension, and (d) Tyndall effect in Ti_3_C_2_ nanosheets suspension., Figure S2: TEM image of the acidified MWCNT., Figure S3: Contact angles of the Ti_3_C_2_-MWCNT membranes., Figure S4: Light reflection of the (a,b) pristine Ti_3_C_2_ and (c,d) T-50C membranes, Figure S5: (a,b,d,e) Cross-section and (c,f) top-view SEM images of the T-20C/T-80C membranes, Table S1: Solar desanlination performance of various 2D materials-based photothermal membranes.
