# Supplementary material for: 2D/1D MXene/MWCNT Hybrid Membrane-Based Evaporator for Solar Desalination
Source: Materials (Basel). 2022 Jan 25;15(3):929. doi: 10.3390/ma15030929 (PMC8839898; doi:10.3390/ma15030929)
Supplement: Supplementary file 1 [file materials-15-00929-s001.zip › materials-1552906-supplementary.pdf]

materials

Supplementary

2D/1D MXene/MWCNT Hybrid Membrane-Based Evaporator for Solar Desalination

Yawei Yang *, Yan Han, Jianqiu Zhao and Wenxiu Que *

Calculation of the solar-to-vapor conversion efficiency

The solar-to-vapor conversion efficiency (η) is estimated by

$$\eta = (m_{light} - m_{dark})h_{LV}/I \quad (S1)$$

$$h_{LV} = Q + L_v \quad (S2)$$

$$L_v = 1.91846 \times 10^3 \times [T_i/(T_i - 33.91)]^2 \quad (S3)$$

$$Q = C\,(T_i - T_s) \quad (S4)$$

where m_{light} and m_{dark} is the evaporation rate under light and dark conditions, h_{LV} is the total enthalpy of sensible heat (Q, J/g) and liquid-vapor phase change (L_v, J/g), I is power density of solar illumination (1 kW/m^2), and C is the specific heat of water (4.2 J/g K). L_v is dependent on the water/air interface temperature (T_i). Q is determined by the temperature difference between the water/air interface (T_i) and source water (T_s).

In this case, T_s = 25 °C. T_i = 36 and 38 °C for Ti_3C_2 and Ti_3C_2-MWCNT membranes, respectively. Thus,

$h_{LV} = L_v + Q$ = 2416.8 + 46.2 = 2463.0 J/g for Ti_3C_2 membrane

$h_{LV} = L_v + Q$ = 2420.6 + 54.6 = 2475.2 J/g for Ti_3C_2-MWCNT membrane

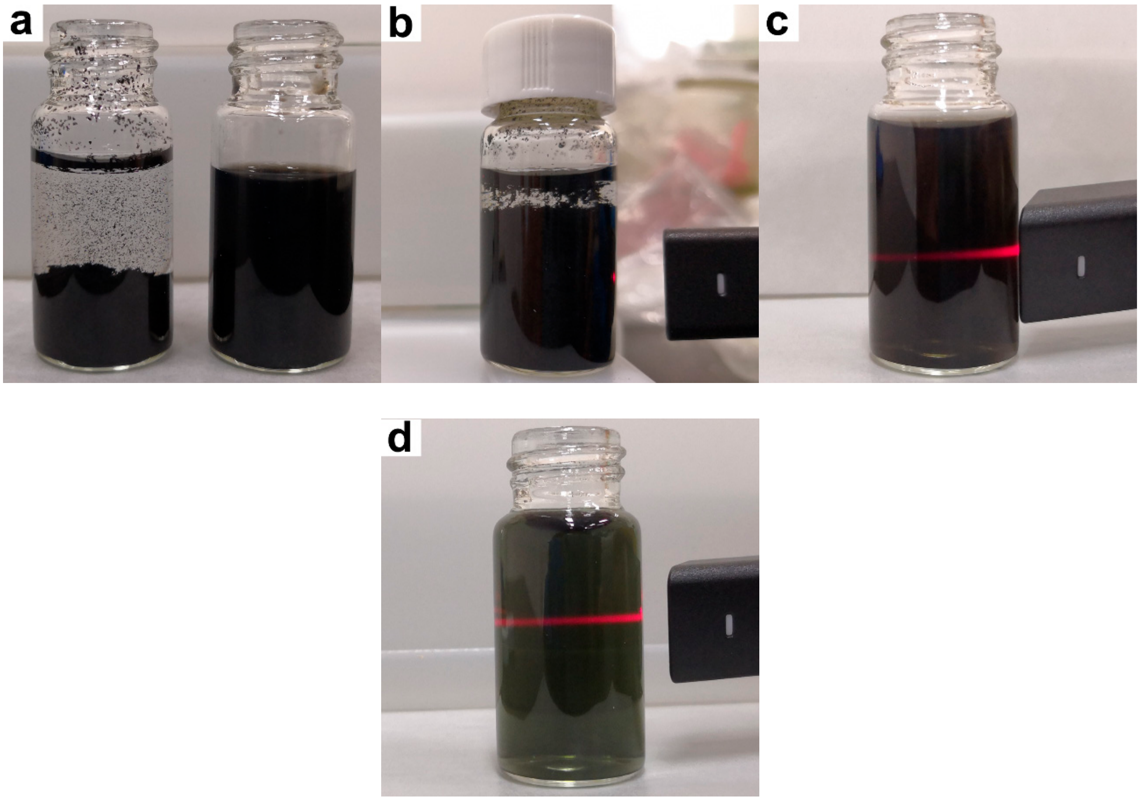

Figure S1. Photographs of (a) the original MWCNT after 3 h of ultrasonic dispersion (left) and well-dispersed acidified MWCNT suspension (right), (b) no Tyndall effect in original MWCNT, (c) Tyndall effect in MWCNT suspension, and (d) Tyndall effect in Ti_3C_2 nanosheets suspension.

Figure S1c,d show that the acidified MWCNT and Ti_3C_2 nanosheets suspensions are homogeneous colloidal dispersions with particle size <100 nm.

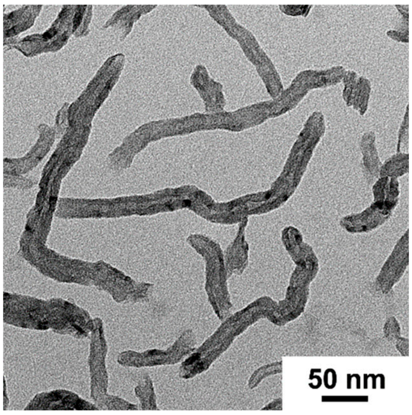

Figure S2. TEM image of the acidified MWCNT.

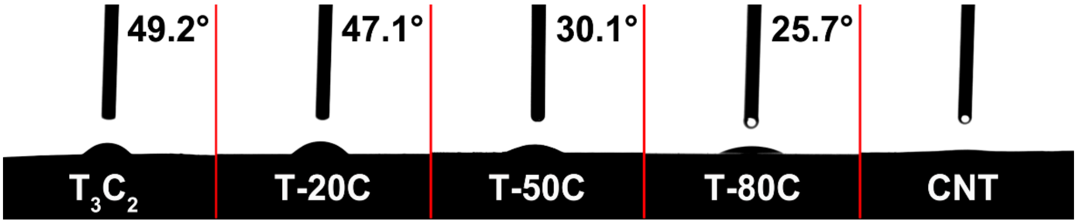

Figure S3. Contact angles of the Ti_3C_2-MWCNT membranes.

The acidified MWCNT (marked as CNT) shows great hydrophilicity mainly due to the adsorbed hydroxyl and carboxyl groups on its surface during acidification. Figure S3 shows that all the Ti_3C_2-MWCNT membranes are hydrophilic, and the hydrophilicity improves with the increasing MWCNT content.

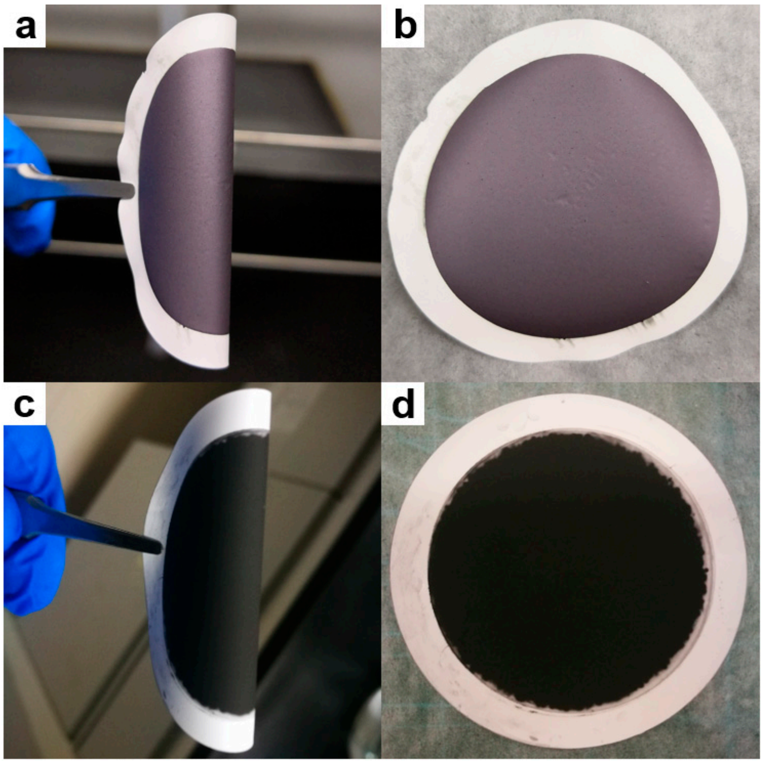

Figure S4. Light reflection of the (a, b) pristine Ti_3C_2 and (c, d) T-50C membranes.

Figure S4a,b show a strong light reflection in the 2D Ti_3C_2 membrane, while Figure 4S (c) and (d) exhibit a significantly reduced light reflection in the T-50C membrane.

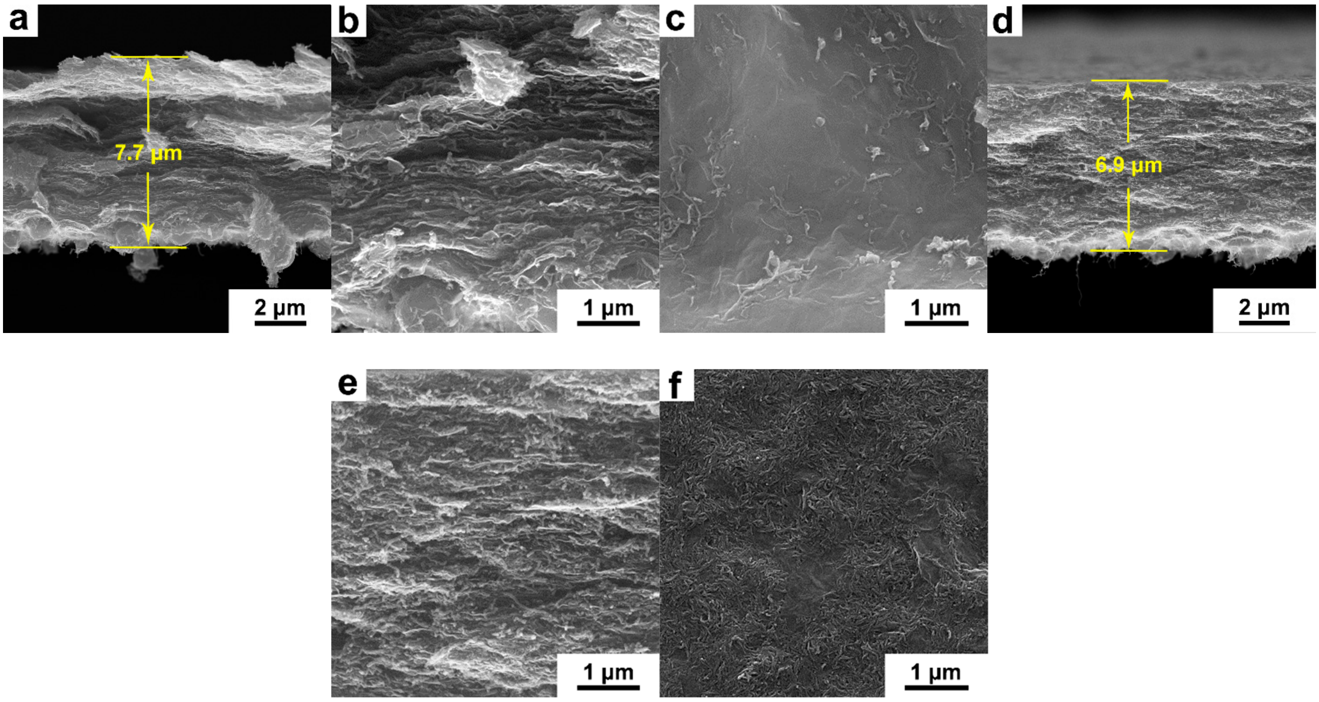

Figure S5. (a, b)/(d, e) Cross-section and (c)/(f) top-view SEM images of the T-20C/T-80C membranes.

Figure S5a,d show that the thickness of T-20C and T-80C are ~7.7 and 6.9 μm, respectively.

Table S1. Solar desanlination performance of various 2D materials-based photothermal membranes.

Sample	Evaporation rate (kg/m² h)	Efficiency (%)	Sun	Salt rejection rate (%)	Ref.
**Ti_3C_2 membrane**	N/A	84	1.0	N/A	*ACS Nano* 2017, 11, 3752
**Ti_3C_2 membrane/ PFDTMS-modified Ti_3C_2 membrane**	1.41/ 1.31	74/ 71	1.0	>99.5	*J. Mater. Chem. A* 2018, 6, 16196
**2D/0D Ti_3C_2/Au membrane**	2.66	83.63	2.0	N/A	*ACS Sustainable Chem. Eng.* 2021, 9, 11372
**2D/2D Ti_3C_2/MoS_2 membrane**	1.36	87.2	1.0	~99.9	*J. Colloid Interface Sci.* 2021, 584, 125
**2D/3D Ti_3C_2/Cu_3BiS_3 membrane**	1.32	90	1.0	>99.9	*ACS Appl. Mater. Interfaces* 2021, 13, 16246
**2D/1D rGO/MWCNT membrane**	1.22	80.4	1.0	N/A	*J. Mater. Chem. A* 2018, 6, 963
**rGO membrane**	0.7163	49.0	1.0	>99.8	*Desalination* 2018, 442, 1
**Ce-MoS_2 membrane**	0.81	75.6	0.76	N/A	*Nano Energy* 2018, 53, 949
**GO membrane**	1.45	80	1.0	>99.9	*PNAS* 2016, 113, 139523
**MoS_2 membrane**	2.50	89.6	1.0	>99.0	*Adv. Mater.* 2020, 32, 2001544
**2D layered $BiInSe_3$ membrane**	1.1	N/A	1.0	>99.9	*J. Mater. Chem. A* 2018, 6, 3869
**2D/2D Ti_3C_2/MWCNT membrane**	**1.55**	**90.8**	**1.0**	**>99.5**	**This work**
